# Normal Values of Hertel Exophthalmometry in a Chinese Han Population from Shenyang, Northeast China

**DOI:** 10.1038/srep08526

**Published:** 2015-02-23

**Authors:** Dan Wu, Xin Liu, Di Wu, Xin Di, Haixia Guan, Zhongyan Shan, Weiping Teng

**Affiliations:** 1 Department of Endocrinology and Metabolism, The Endocrine Institute and The Liaoning Provincial Key Laboratory of Endocrine Diseases, The First Affiliated Hospital of China Medical University, Shenyang, Liaoning Province, 110001, People's Republic of China; 2 Department of Cadre, The Third Affiliated Hospital of Shenyang Medical College (Shenyang 242 Hospital), Shenyang, Liaoning Province, 110034, People's Republic of China; 3 Department of Ophthalmology, Shenyang He Eye Hospital, Shenyang, Liaoning Province, 110001, People's Republic of China

## Abstract

Aims of this study were to determine the normal range of absolute and relative Hertel exophthalmometric values (EVs) in a Chinese Han population. This population-based cross-sectional study consisted of 2010 healthy Han Chinese (1051 females and 959 males) aged between 8–87 years living in Shenyang, Northeast China, including 515 children (aged 8–14 years), 517 teenagers (aged 15–19 years), 582 adults (aged 20–69 years) and 396 elderly (aged 70–87 years). A Hertel exophthalmometer was used by the same physician for the measurement of EV and inter-orbital distance (IOD). For the entire study population, the Hertel EVs ranged from 10 mm to 22 mm; the mean EVs for the left eye (OS) and right eye (OD) were 15.0 ± 1.9 mm and 15.0 ± 2.0 mm, respectively; the upper normal limits of the EVs (mean + 2 SD) for OS and OD were 18.8 mm and 19.0 mm, respectively; the mean relative EV was 0.20 ± 0.43 mm. Age, but not sex, had a significant effect on the EV. We concluded that our study provides normative ophthalmic data in a Chinese Han population. The normal EVs, asymmetry and IOD values have been established for clinical reference.

Many orbital diseases can cause proptosis, including thyroid-associated orbitopathy, tumors, inflammation, head and orbital trauma, and craniofacial abnormalities^1^,^2^,^3^. Upon happening, these diseases are usually manifested as abnormal eye protrusion. For patients in whom orbital diseases are suspected, exophthalmometry is an established method for routine evaluation. The exophthalmometric values (EVs) obtained can either serve as a diagnostic basis or be used to monitor the progress of orbital disease via serial measurements^4^. There are several types of exophthalmometric devices to measure EVs, Hertel and Luedde measure the distance of the corneal apex from the level of the lateral orbital rim while Naugle measures the relative difference between each eye^5^. Among them, the most widely used device nowadays is Hertel exophthalmometry^6^,^7^. It allows measurements of the distance between the two lateral orbital rims (i.e. inter-orbital distance, IOD) and the vertical distance of corneal apex to the frontal plane. This method can measure both eyes simultaneously using a mirror system and a superimposed millimeter scale^8^.

According to literatures^6^,^7^,^9^, the absolute EV represents the degree of protrusion when compared with a given standard, hence it is very useful for diagnosing bilateral proptosis. The relative EV reflects the asymmetry of protrusion between two eyes and therefore, relative exophthalmometry is beneficial for the diagnosis of unilateral proptosis. The comparative EV reflects a change in the amount of protrusion in follow-up examinations, which can document the progression of the condition causing the proptosis.

The current literature clearly demonstrates heterogeneity in EV ranges and their correlates among different populations^4^,^8^,^9^,^10^,^11^,^12^,^13^,^14^,^15^,^16^,^17^,^18^,^19^. Therefore, when evaluating the results of measurements, the normal EV range in a specific population must be taken into consideration. However, because only a few small, underpowered studies have reported normative exophthalmometry data for Chinese population^12^,^13^,^14^, proptosis diagnosis in Mainland China is currently based on data from other countries and races. As a result, we believe it is important to define a normal EV in a large Chinese population.

In an attempt to provide normative ophthalmic data in a Chinese Han population, a large, population-based cross-sectional study was conducted in Shenyang, Northeast China. Demographical and ophthalmic data collected in this study allowed us to determine the normal range of absolute and relative EVs, to analyze the impact of age, sex and IOD values on EVs, and to compare these EVs with previously reported EVs obtained from several populations with different racial backgrounds as well as from two Chinese cohorts from Hong Kong and Taiwan.

## Results

A total of 2010 healthy Han Chinese (1051 females and 959 males) aged between 8–87 years living in Shenyang, Northeast China participated in this study. The participants included 515 children (aged 8–14 years), 517 teenagers (aged 15–19 years), 582 adults (aged 20–69 years) and 396 elderly (aged 70–87 years). There was no significant sex difference among the various age groups (*P* > 0.05).

The EV data for the studied population is presented in Table 1. The Hertel EVs for the entire study population ranged from 10 mm to 22 mm, and the mean was 15.0 ± 1.9 mm for the left eye (OS) and 15.0 ± 2.0 mm for the right eye (OD). The upper normal limit of EV (mean + 2 SD) for OS and OD were as follows: 16.9 mm and 16.7 mm, respectively, in children; 18.1 mm and 18.3 mm, respectively, in teenagers; 19.3 mm and 19.3 mm, respectively, in adults; 19.7 mm and 19.9 mm, respectively, in the elderly; and 18.8 mm and 19.0 mm, respectively, in the entire study population. The EVs of OS and OD strongly correlated with each other (r = 0.949 – 0.998, *P* < 0.001) in all age groups. No statistically significant differences were indicated between the EVs of OS and OD in any age group. The mean relative EVs (absolute difference between bilateral EVs) were as follows: 0.01 ± 0.09 mm in children; 0.25 ± 0.44 mm in teenagers; 0.23 ± 0.45 mm in adults; 0.32 ± 0.53 mm in the elderly, and 0.20 ± 0.43 mm in the entire study population. Asymmetric measurements occurred in 370 (18.4%) subjects; 348 subjects showed 1 mm of difference, and 22 subjects exhibited 2 mm of difference.

**Figure 1:**
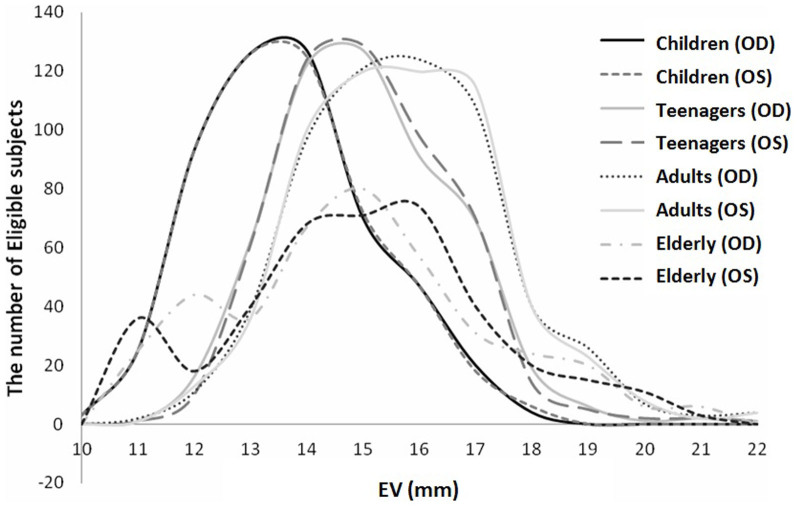
Distributions of EVs in Han Chinese Subjects of Different Age Groups. EV, exophthalmometric value; OS, left eye; OD, right eye.

**Figure 2:**
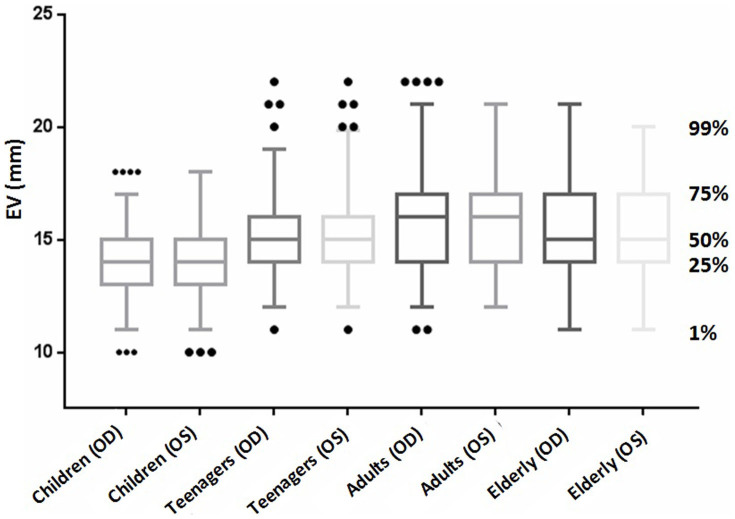
Box-plot Histograms of EVs in Han Chinese Subjects of Different Age Groups. EV, exophthalmometric value; OS, left eye; OD, right eye.

Figure 1 shows the distribution of EVs stratified for age. The data appeared to be approximately normally distributed. It is evident that the curves shifted to the right from children to adults, then reversed slightly leftward in the elderly. As shown in Table 1 and Figure 2, children had the lowest mean EV, whereas adults had the highest. When the EV results were compared between males and females, a statistically significant difference could not be found (*P* > 0.05), although the mean EV for males was slightly greater than that for females in each age group (Table 2).

Increasing age had a significant positive correlation with the EV in children and teenagers and a significant negative correlation in the elderly (Table 3, *P* < 0.01). The EV was positively correlated with the IOD value in all age groups (*P* < 0.001). The equations in Table 4 are useful for calculating the expected EV (y) using IOD values (x) for Chinese Han subjects at each age group (simple linear regression analysis).

## Discussion

The identification of eye protrusion relies not only on accurate measurements but also on normal EV reference values^14^. Presently, there are many techniques to evaluate exophthalmometry. Radiological imaging such as magnetic resonance imaging (MRI) and computerized tomography (CT) are considered as the most accurate methods of determining exophthalmos and are not affected by the facial asymmetry and increased soft tissue volume^8^,^20^. However, there are certain caveats, including the high expense, the long exposure to radiation (from CT scans), and lack of suitability for epidemiological investigations involving a large number of individuals^8^,^19^. Although several limitations have been identified for its use, the Hertel exophthalmometer is still the most widely used instrument for measuring exophthalmos, due to its easy operation, low expense and portable convenience^21^,^22^,^23^.

In this study, we used a Hertel exophthalmometer to investigate normal EVs in a Chinese Han population from the northeastern region of China. This population consisted of 2010 subjects, including children, teenagers, adults and elderly. Because previous studies have reported that the results of EVs conducted on the same individual tend to vary between researchers^21^,^22^,^23^,^24^,^25^,^26^, we tried to minimize this unwanted interference by having all the measurements done by the same physician. We determined that the mean EV for this Chinese population to be approximately 15 mm. In comparison with previous studies that were performed on normal subjects of different races (Table 5), the mean EV for these Chinese adults seemed to be greater than that of the Turkish^8^,^19^ and close to that of the Mexican^17^ and Iranian^9^, but lower than other non-Chinese populations^4^,^10^,^16^,^18^,^27^,^28^. Notably, the mean EV obtained from the present study is much greater than the 12–14 mm written in *Ophthalmology*, a widely recognized Chinese medical reference textbook^29^. If the upper normal limit was defined as 2 standard deviations above the mean^9^,^14^, for the Chinese Han subjects obtained from our study, the upper normal limit of the EVs for OS and OD were 16.9 mm and 16.7 mm in children, 18.1 mm and 18.3 mm in teenagers, 19.3 mm and 19.3 mm in adults, 19.7 mm and 19.9 mm in the elderly, 18.8 mm and 19.0 mm in the entire study population. These values are lower than those reported by Migliori et al.^10^ and Barretto et al.^27^, which were 20.1–20.4 mm for white women, 21.7–22.3 mm for white men, 20.2–23.0 mm for black women, and 22.8–24.7 mm for black men, but close to the values reported by investigators in Korea^30^ and Japan^31^, which varied from 17.7 to 18.3 mm. Our results confirmed that the EV reference range does differ by race, possibly due to discrepancies in facial structures and ocular anatomy. This inter-racial difference should be given more attention. When diagnosing eye proptosis, clinicians should be aware that using data from other countries and races to evaluate their patient is not appropriate.

Tsai et al.^14^ and Quant et al.^12^,^13^ performed similar exophthalmometric studies in Taiwan and Hong Kong, respectively. Although they both conducted investigations in Chinese populations, results from their studies were markedly different from each other. Our mean EV results are higher than those in Tsai's study but lower than those in Quant's studies (Table 5). Compared to Tsai and Quant's studies, we had a much larger sampling size of 2010 subjects and covered a wider age range (8–87 years). Besides, our study was performed on a northern Chinese population whereas the other two research groups studied subjects in the south. Thus, variation from these Chinese studies might be due to the sample size, exclusion criteria and regional differences. As China is geographically vast, we believe our results are more suitable for application to the Chinese population living in the northern regions of China.

Apart from race, sex and age were also considered as factors of variation in the normal range of EVs; however, there were no consistent conclusions across studies. Some studies have shown a significant difference between the sexes, especially in black and white Americans^10^, but no difference was observed in our study. With respect to age, previous studies have demonstrated a general trend of EV increases during growth (first two decades of life), no change or a linear decrease during adulthood (third to sixth decade of life) and decreases in later decades (seventh decade onwards)^4^,^10^,^32^,^33^,^34^,^35^, but Bilen et al.^8^ failed to confirm this trend in a Turkish study. Thus, we took advantage of this large series of subjects to also analyze the relationship between age and EVs. Our results seem to conform to the above-described pattern. In particular, compared with other age groups, children had significantly lower EVs. Accordingly, we believed that age variation should be taken into account when designing and interpreting a normal EV study; in addition, a specific reference range for evaluating proptosis should be considered for children in our clinical practice.

In our study, EVs were found to be consistent between left and right eyes with only a minority having a 1–2 mm difference. The maximum relative EV of our subjects was 2 mm. In the majority of reports from different ethnic backgrounds, the relative protrusion value is not more than 2 mm^10^,^19^,^26^,^30^. Therefore, we agree with other researches^9^ that relative ocular protrusion value of up to 2 mm in the absence of other pathological findings may have little clinical significance; on the other hand, an asymmetry of more than 2 mm should be noted during medical check-ups.

In conclusion, because reports of normative exophthalmometry data for a Chinese population are insufficient and inconsistent, our study is of value. Based on the present findings, we established normal EVs, asymmetry and IOD values in a population with Han ethnicity, providing a diagnostic reference for exophthalmos. The EV significantly increases with increasing age in children and teenagers and decreases in later decades. Sex does not have a significant effect on the EV. The EVs are consistent between left and right eyes with a maximum relative EV of 2 mm. This study was conducted in the northeastern part of China, so it is important to emphasize that our findings may only be reflective of the normal values in the Chinese population of this region. As China is geographically vast, an inter-area variation in the normal range of EVs may exist. Consequently, well-designed, larger and multi-center studies are necessary in the future to provide more informative data on the whole Chinese population.

## Methods

This investigation was a population-based, cross-sectional epidemiological study. It was conducted in Shenyang City, Northeast China. The demographics in this city were representative of the population in northern China. Because the majority of inhabitants (> 90%) in Shenyang and in the Chinese mainland are of the Han ethnicity, the present study was conducted in Han Chinese.

There were nine districts in Shenyang City. Using simple random sampling (SRS), three districts were selected for the recruitment of children (defined as being 7–14 years old), teenagers (defined as being 15–19 years old) and adults (defined as being 20–69 years old) /elderly (defined as being ≥ 70 years old), respectively. Then, from the list of primary schools in the designated district for recruiting children, two schools were randomly selected (SRS); from the list of high schools and colleges in the designated district for recruiting teenagers, one high school and one college were randomly selected (SRS); from the list of communities in the designated district for recruiting adults/elderly, one community (including local factories, offices and residential quarters) was randomly selected (SRS). Once the schools and community were chosen, we advertised and promoted the study prior to commencement of the study, so that those who were qualified could volunteer to participate. In each facility, an appropriate examination site was set during the study. The exclusion criteria included a history of systemic or local diseases that may affect the orbit or the orbital cavity, including orbital tumors, inflammation, vascular disorders, trauma, surgery, endocrine system disease, buphthalmos and craniofacial malformations. Also excluded were individuals with myopia or hyperopia of more than 3 diopters equivalent sphere because we wanted to rule out any influence of changed axial length on the exophthalmometry reading^19^.

Exophthalmometry was measured using an accurately calibrated Hertel exophthalmometer (Keeler Instruments Inc., Broomall, PA, USA). The detailed exophthalmometry procedures were the same as reported previously^9^. The EV was measured to the nearest 1 mm. The left eye and then right eye were measured in all subjects. The IOD value was recorded to the nearest 1 mm.

In order to avoid variations between researchers, in the present study, all the measurements were performed by the same physician (Dr. Liu X.). To test the accuracy of measurements conducted by this physician, an experienced ophthalmologist (Dr. Di X.) used the same device to re-assess 100 subjects. Bland- Altman analysis showed a clinically acceptable agreement between the two doctors (see Supplementary Fig. S1 online). Moreover, paired *t*-tests did not show significant inter-observer difference in IOD values (*P* = 0.085) and EVs (for OS, *P* = 0.343; for OD, *P* = 0.299). Taken together these results suggest that measurements done by this physician are reliable.

This study was approved by the Medical Ethics Committee of the First Affiliated Hospital of China Medical University and was conducted in accordance with approved guidelines and regulations. Written consent was obtained from all subjects.

The data are presented as the mean ± standard deviation (SD), range, and the 95^th^ /97.5^th^/99^th^ percentiles (for EVs only). Statistical analysis was using the Student *t*-test and ANOVA. The upper limit of normal exophthalmometry was defined by taking the mean + 2SD^9^,^14^. The relationship between the EV and IOD was determined by linear regression analysis. A *P* value less than 0.05 was considered statistically significant. Statistical analysis was performed using SPSS software (version 16.0, Chicago, IL, USA).

## Author Contributions

Dan W., X.L. and X.D. carried out the study. Dan W., X.L. and Di W. wrote the main manuscript. X.D. prepared the tables 1–4. H.G. designed and funded the study, prepared the table 5 and figures, and revised the manuscript. Z.S. and W.T designed the study. All authors reviewed the manuscript.

## Supplementary Material

Supplementary Information Supplementary Fig. S1

## Figures and Tables

**Table 1:** EVs and IOD Values in Han Chinese Subjects of Different Age Groups

Age Group (no.)	Age (mean ± SD, yr)	EV (mean ± SD, mm)	Percentiles of EV (95^th^/97.5^th^/99^th^, mm)	Range of EV (mm)	Absolute Difference between Bilateral EVs (mean ± SD, mm)	IOD (mean ± SD, mm)	Range of IOD (mm)
Children (n = 515)	10.6 ± 1.1	13.7 ± 1.6 (OS) *	16.0 / 17.0 / 17.8	10–18	0.01 ± 0.09*	100.2 ± 5.0*	87–114
		13.7 ± 1.5 (OD) *	16.0 / 17.0 / 17.0	10–18			
Teenagers (n = 517)	16.7 ± 1.5	15.1 ± 1.5 (OS)	17.0 / 18.0 / 19.0	11–22	0.25 ± 0.44	108.5 ± 3.9	100–121
		15.1 ± 1.6 (OD)	18.0 / 18.0 / 19.0	11–22			
Adults (n = 582)	39.1 ± 14	15.7 ± 1.8 (OS) #	19.0 / 19.0 / 20.2	11–22	0.23 ± 0.45	111.0 ± 4.0	90–122
		15.7 ± 1.8 (OD) #	19.0 / 19.0 /21.0	11–22			
Elderly (n = 396)	76.6 ± 4.1	15.3 ± 2.2 (OS)	19.0 / 20.0 / 20.0	11–22	0.32 ± 0.53	110.8 ± 4.8	94–120
		15.3 ± 2.3 (OD)	19.0 / 20.0 / 21.0	11–22			
Total (n = 2010)	33.4 ± 25.4	15.0 ± 1.9 (OS)	18.0 / 19.0 / 20.0	10–22	0.20 ± 0.43	107.6 ± 6.3	87–122
		15.0 ± 2.0 (OD)	18.0 / 19.0 / 20.0	10–22			

Abbreviations: EV, exophthalmometric value; IOD, inter-orbital distance; OS, left eye; OD, right eye; SD, standard deviation.

^*^, ^#^: compared with other age groups, *P* < 0.01.

**Table 2:** Comparison of EVs between Females and Males

Age Group (no.)	Gender Group (no.)	Age (mean ± SD, yr)	EV (mean ± SD, mm)	
			OS	OD
Children (n = 515)	Female (n = 256)	10.6 ± 1.5	13.6 ± 1.5	13.6 ± 1.5
	Male (n = 259)	10.6 ± 1.1	13.8 ± 1.6	13.8 ± 1.6
Teenagers (n = 517)	Female (n = 275)	16.6 ± 1.4	14.9 ± 1.5	15.0 ± 1.5
	Male (n = 242)	16.9 ± 1.5	15.2 ± 1.7	15.2 ± 1.6
Adults (n = 582)	Female (n = 295)	38.6 ± 13.8	15.7 ± 1.8	15.8 ± 1.8
	Male (n = 287)	39.6 ± 14.2	15.8 ± 1.8	15.7 ± 1.8
Elderly (n = 396)	Female (n = 225)	76.3 ± 4.3	15.2 ± 2.3	15.2 ± 2.2
	Male (n = 171)	77.0 ± 3.9	15.5 ± 2.2	15.6 ± 2.2
Total (n = 2010)	Female (n = 1051)	34.0 ± 25.7	14.9 ± 2.0	15.0 ± 1.9
	Male (n = 959)	32.7 ± 25.0	15.0 ± 2.0	15.1 ± 1.9

Abbreviations: EV, exophthalmometric value; OS, left eye; OD, right eye; SD, standard deviation.

**Table 3:** Correlation of EVs with Age and IOD values

Age Group (no.)		Correlation of EV with Age		Correlation of EV with IOD	
		R	*P*	R	*P*
Children (n = 515)	OS	0.114	0.010	0.607	<0.001
	OD	0.114	0.010	0.606	<0.001
Teenagers (n = 517)	OS	0.207	<0.001	0.365	<0.001
	OD	0.217	<0.001	0.372	<0.001
Adults (n = 582)	OS	0.038	0.366	0.318	<0.001
	OD	0.027	0.511	0.323	<0.001
Elderly (n = 396)	OS	−0.207	<0.001	0.308	<0.001
	OD	−0.199	<0.001	0.324	<0.001
Total (n = 2010)	OS	0.230	<0.001	0.537	<0.001
	OD	0.223	<0.001	0.536	<0.001

Abbreviations: EV, exophthalmometric value; IOD, inter-orbital distance; OS, left eye; OD, right eye.

**Table 4:** Equations for Calculating the EV (y) and IOD Values (x) for Chinese Han Subjects

Age Group	N	OS		OP	
		Equations	F value	Equations	F value
Children	515	y = 0.188x − 5.110	298.9	y = 0.187x − 4.994	297.8
Teenagers	517	y = 0.142x − 0.369	79.1	y = 0.150x − 1.204	82.6
Adults	582	y = 0.140x + 0.150	65.4	y = 0.144x − 0.235	67.5
Elderly	396	y = 0.143x − 0.475	41.4	y = 0.153x − 1.638	46.1

Abbreviations: EV, exophthalmometric value; IOD, inter-orbital distance; OS, left eye; OD, right eye.

**Table 5:** Comparison of Hertel EVs among Subjects of Different Ethnic Groups

Study	Ethnic Group	Age	No.	Eye	EV Mean ± SD	IOD Mean ± SD
This Study	Chinese Han (Northeast China)	8–14	515	OU	13.7 ± 1.6	100.2 ± 5.0
		15–19	517	OU	15.1 ± 1.6	108.5 ± 3.9
		20–69	528	OU	15.7 ± 1.8	111.0 ± 4.0
		70–87	398	OU	15.3 ± 2.2	110.8 ± 4.8
Quant et al. [12]	Chinese (Hong Kong)	18–60	243	OD	16.7 ± 1.9	M: 101.5 ± 4.3
				OS	16.6 ± 1.8	F: 97.9 ± 4.1
Quant et al. [13]	Chinese (Hong Kong)	7–11	232	OU	15–17	90–97
Tsai et al [14]	Chinese (Taiwanese)					
	M	59.4 (mean)	188	OU	13.97 ± 2.26	106.45 ± 4.12
	F	49.0 (mean)	231	OU	13.86 ± 2.39	105.31 ± 4.36
Migliori et al [10]	White American	18–91				
	M		127	OD	16.55 ± 2.57	98.3 ± 3.9
				OS	16.47 ± 2.60	
	F		200	OD	15.46 ± 2.34	95.4 ± 3.8
				OS	15.36 ± 2.33	
	African American	18–91				
	M		113	OD	16.55 ± 2.57	101.4 ± 3.7
				OS	16.47 ± 2.60	
	F		241	OD	15.46 ± 2.34	98.5 ± 3.5
				OS	15.36 ± 2.33	
Cole et al [7]	White American	42.8 (mean)	57			
	M			OU	17.34 ± 2.52	104.8 ± 5.9
	F			OU	17.47 ± 2.26	104.3 ± 7.2
	African American	42.8 (mean)	148			
	M			OU	18.57 ± 2.59	106.6 ± 6.6
	F			OU	19.23 ± 2.35	104.9 ± 6.8
Dunsky et al [15]	African American	19–80				
	M		170	OU	18.20 ± 2.97	99.86 ± 3.57
	F		139	OU	17.46 ± 2.64	95.70 ± 3.44
Dijkstal et al [16]	American	9.6 (mean)				
	M	1–17 (range)	322	NA	15.1 ± 1.8	92.5 ± 5.9
	F		351	NA	15.0 ± 2.0	91.5 ± 5.1
Bolanos Gil deMontes et al [17]	Mexican	36 (mean)				
	M		116	NA	15.18 ± 2.16	97.78 ± 3.97
	F		185	NA	14.82 ± 1.98	94.33 ± 1.98
Fledelius et al [18]	White European					
	M	5–10	50	OU	13.7 ± 1.4	88.3 ± 3.8
		11–19	71	OU	15.0 ± 1.7	94.1 ± 4.0
		19–80	102	OU	16.5 ± 2.3	97.8 ± 3.5
	F	5–10	53	OU	13.6 ± 1.7	85.6 ± 3.8
		11–19	77	OU	15.1 ± 1.7	92.2 ± 3.7
		19–80	101	OU	16.0 ± 1.7	93.7 ± 3.6
Beden et al [19]	Turkish					
	M	49.9 (mean)	960	OD	13.49 ± 2.49	NA
				OS	13.38 ± 2.48	
	F		1507	OD	13.27 ± 2.40	NA
				OS	13.18 ± 2.42	
Bilen et al [8]	Turkish	3–80				
	M		240	OU	13.49 ± 2.6	NA
	F		240	OU	13.44 ± 2.6	NA
Chan et al [4]	Sri Lankan					
	M	57.9 (mean)	532	OU	16.66	NA
	F	56.4 (mean)	809	OU	15.27	NA
Kashkouli et al [9]	Iranian	6–12	289	OD	14.3 ± 1.8	100.0 ± 3.9
				OS	14.1 ± 1.8	
		13–19	319	OD	15.3 ± 1.9	107.7 ± 4.2
				OS	15.1 ± 1.9	
		20–70	455	OD	14.8 ± 2.3	108.7 ± 4.0
				OS	14.6 ± 2.3	
Osuobeni et al [11]	Arabic (M)	6–12	406	OD	15.4 ± 1.6	95.7 ± 3.4
				OS	15.2 ± 1.6	

Abbreviations: EV, exophthalmometric value; IOD, inter-orbital distance; M, male; F, female; OS, left eye; OD, right eye; OU, average of both eyes; SD, standard deviation; NA, not available.
