# Supplementary material for: Normal Values of Hertel Exophthalmometry in a Chinese Han Population from Shenyang, Northeast China
Source: Sci Rep. 2015 Feb 23;5:8526. doi: 10.1038/srep08526 (PMC4336939; doi:10.1038/srep08526)
Supplement: Supplementary Information — Supplementary Fig. S1 [file srep08526-s1.doc]

**Supplementary information**

**Title:** Normal Values of Hertel Exophthalmometry in a Chinese Han Population from Shenyang, Northeast China

**Authors:** Dan Wu^1, 2,#^, Xin Liu^1,#^, Di Wu^1^ , Xin Di^3^ , Haixia Guan^1^, *, Zhongyan Shan^1^, Weiping Teng^1^

**Affiliations:** ^1^ Department of Endocrinology and Metabolism, The Endocrine Institute and The Liaoning Provincial Key Laboratory of Endocrine Diseases, The First Affiliated Hospital of China Medical University, Shenyang, Liaoning Province, 110001, People’s Republic of China.

^2^ Department of Cadre, The Third Affiliated Hospital of Shenyang Medical College (Shenyang 242 Hospital), Shenyang, Liaoning Province, 110034, People’s Republic of China.

^3^ Department of Ophthalmology, Shenyang He Eye Hospital, Shenyang, Liaoning Province, 110001, People’s Republic of China.

^#^ Both authors contributed equally to this work. * Corresponding author.


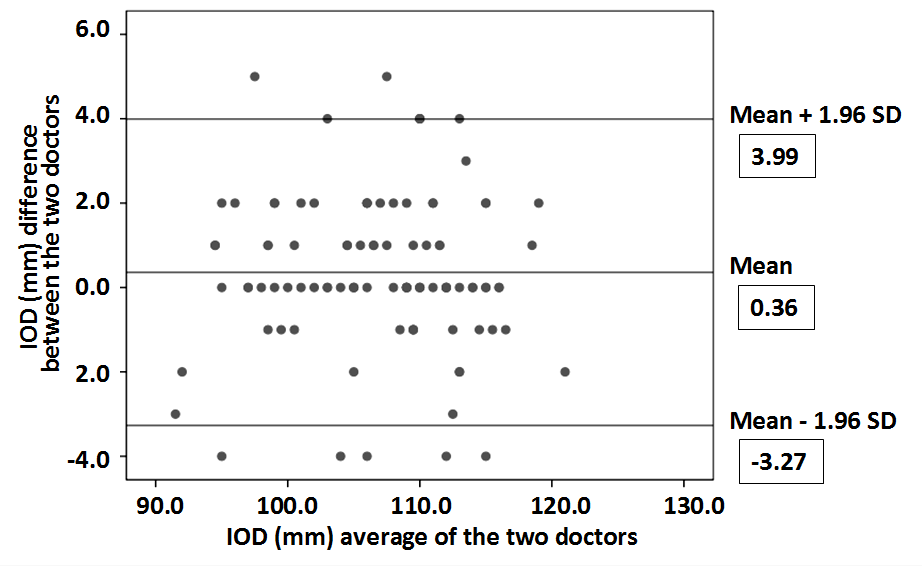

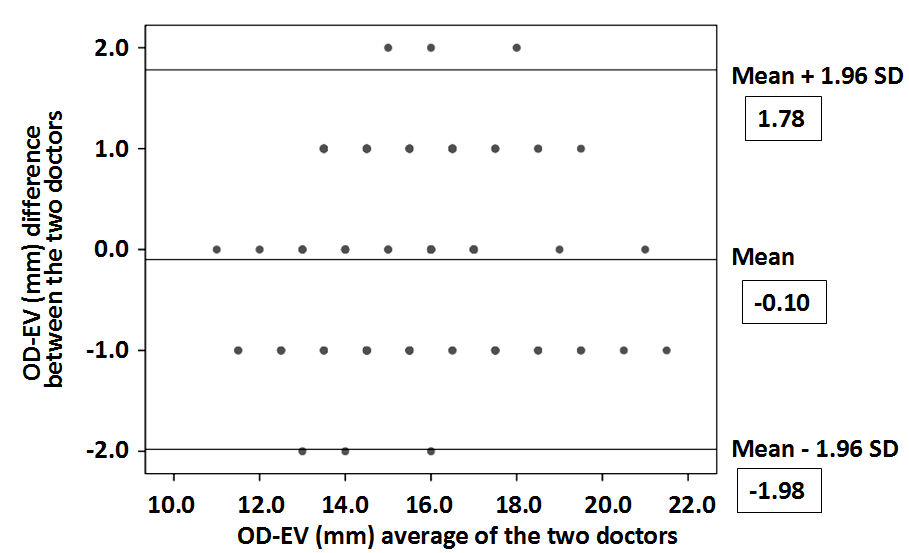

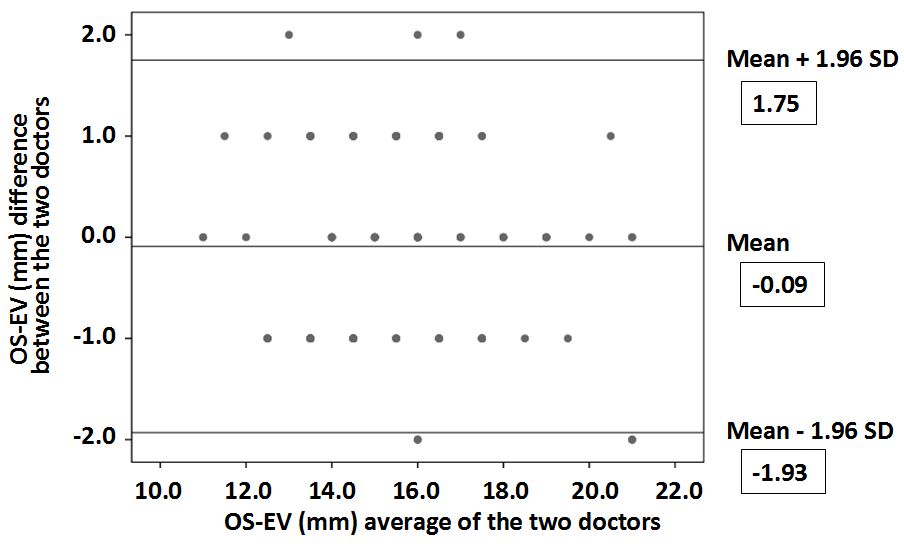


**Supplementary Fig. Sl Inter-observer Reliability between the Physician and the Experienced Ophthalmologist Displayed by Bland-Altman Plot.** IOD, inter-orbital distance; EV, exophthalmometric value; OS, left eye; OD, right eye; SD, standard deviation.
